# Active and prospective latent tuberculosis are associated with different metabolomic profiles: clinical potential for the identification of rapid and non-invasive biomarkers

**DOI:** 10.1080/22221751.2020.1760734

**Published:** 2020-06-02

**Authors:** A. Albors-Vaquer, A. Rizvi, M. Matzapetakis, P. Lamosa, A. V. Coelho, A. B. Patel, S. C. Mande, S. Gaddam, A. Pineda-Lucena, S. Banerjee, L. Puchades-Carrasco

**Affiliations:** a Drug Discovery Unit, Instituto de Investigación Sanitaria La Fe, Hospital Universitario y Politécnico La Fe, Valencia, Spain; b Department of Biochemistry, School of Life Sciences, University of Hyderabad, Hyderabad, India; c ITQB NOVA, Oeiras, Portugal; d CSIR-CCMB, Hyderabad, India; e National Centre For Cell Science, Pune, India; f Council of Scientific and Industrial Research, New Delhi, India; g Department of Immunology, Bhagwan Mahavir Medical Research Center, Hyderabad, India; h Department of Genetics, Osmania University, Hyderabad, India; i Molecular Therapeutics Program, Centro de Investigación Médica Aplicada, University of Navarra, Pamplona, Spain

**Keywords:** Biomarker, latent tuberculosis, metabolomics, NMR, tuberculosis

## Abstract

Although 23% of world population is infected with *Mycobacterium tuberculosis* (*M. tb*), only 5–10% manifest the disease. Individuals surely exposed to *M. tb* that remain asymptomatic are considered potential latent TB (LTB) cases. Such asymptomatic *M. tb*.-exposed individuals represent a reservoir for active TB cases. Although accurate discrimination and early treatment of patients with active TB and asymptomatic *M. tb*.-exposed individuals are necessary to control TB, identifying those individuals at risk of developing active TB still remains a tremendous clinical challenge. This study aimed to characterize the differences in the serum metabolic profile specifically associated to active TB infected individuals or to asymptomatic *M. tb*.-exposed population. Interestingly, significant changes in a specific set of metabolites were shared when comparing either asymptomatic house-hold contacts of active TB patients (HHC-TB) or active TB patients (A-TB) to clinically healthy controls (HC). Furthermore, this analysis revealed statistically significant lower serum levels of aminoacids such as alanine, lysine, glutamate and glutamine, and citrate and choline in patients with A-TB, when compared to HHC-TB. The predictive ability of these metabolic changes was also evaluated. Although further validation in independent cohorts and comparison with other pulmonary infectious diseases will be necessary to assess the clinical potential, this analysis enabled the discrimination between HHC-TB and A-TB patients with an AUC value of 0.904 (confidence interval 0.81–1.00, *p*-value < 0.0001). Overall, the strategy described in this work could provide a sensitive, specific, and minimally invasive method that could eventually be translated into a clinical tool for TB control.

## Introduction

The bacterium *Mycobacterium tuberculosis* (*M. tb*) is the underlying cause of tuberculosis (TB), one of the most devastating infectious diseases. The World Health Organization (WHO) estimates that there are about 10.4 million new cases and 1.8 million deaths from TB each year [1]. Additionally, it is estimated that currently one quarter of the world's population is latently infected with *M. tb* [2]. *M. tb exposed* individuals that remain asymptomatic are generally considered potential latent TB (LTB) cases [3,4]. LTB infection is characterized by a persistent immune response to *M. tb* in the absence of clinical, radiological and microbiological evidences. Only 5–15% of the patients with LTB infection develop active TB disease during their lifetime, being a reservoir of new active TB cases [1,5,6]. Besides drug resistance, co-epidemic with HIV, early diagnosis and failure to identify asymptomatic yet infective cases, are some of the principal concerns in controlling TB. TB diagnosis, till date, banks on medical history and conventional methods, such as tuberculin skin test (TST), chest X-rays, and bacteriological examination. TST and *M. tb* specific interferon-gamma (IFN-γ) release assays (IGRAs) are still the main tools used for the diagnosis of TB infection. Although the newer IGRAs show some improvements over TST [7,8], neither of these diagnostic tests can identify *M. tb.* – exposed individuals that could be LTB cases, nor differentiate between asymptomatic cases and active TB [9]. Indeed, the World Health organization (WHO) strongly recommends that neither IGRAs nor TST should be used in high TB-burden, low – and middle-income countries for screening of LTB cases or for the identification of individuals at risk of developing active TB [10]. Furthermore, WHO strongly recommends that IGRAs should not replace TST in these countries for the screening of LTB cases. Since the reactivation of TB can be prevented by pharmacological treatment [11], accurate discrimination of TB exposed population and early treatment of asymptomatic subjects exposed to TB germ (LTB) and patients with active TB infection are necessary to limit TB. Thus, identifying individuals at risk of developing active TB would have a tremendous impact in TB control [12,13].

In this context, metabolomics approaches have shown great potential for the identification on new clinically relevant potential biomarkers. Metabolic signatures have proven their value in several diseases, such as cancer [14–17], gynaecological diseases [18], hypertension [19] and insulin resistance [20]. In contrast, fewer studies have specifically addressed the metabolomic alterations that occur in infectious diseases [21–23]. Metabolomics provides a promising tool particularly suited for the identification of non-invasive biomarkers for diagnosing and monitoring patients. In this study, we hypothesized that active TB patients, asymptomatic *M.tb.*-exposed potential LTB cases and healthy individuals exhibit distinct serum metabolic signatures that can be characterized by high-resolution nuclear magnetic resonance (^1^H-NMR) spectroscopy. Thus, the aim of this study was to investigate the feasibility of identifying a metabolic signature in serum of TB patients that could facilitate a better understanding of the biochemical changes involved in the progression from possible LTB to active TB.

## Methods

### Study cohort

In this study, a total of 80 subjects were recruited after taking ethical committee clearances (ECR/450/Inst/AP2013, ECR/450/Inst/AP/20131RR-16 and UH/IEC/2014/33) and written consents from the subjects. The subjects were classified as clinically healthy volunteers (HC, *n*=35), active TB patients (A-TB, *n*=15) or TB patients’ household contacts (HHC-TB, *n*=30). TB patients and household contacts were recruited at Mahavir Hospital and Research Centre (MHRC), Hyderabad, India. Healthy volunteers were recruited at Health Centre, University of Hyderabad. A-TB patients were identified as per Revised National Tuberculosis Control Programme (RNTCP) guidelines, Government of India, with confirmed diagnosis from sputum, culture, Mantoux test and chest X-ray in patients. Although A-TB patients included in the study had different acid-fast bacillus (AFB) smear status, all of them were confirmed to be *M. tb*. culture positive. Household contacts of the respective A-TB patients were those who resided in-house of the patient during a 3 months period for at least seven consecutive days prior to the diagnosis of TB. Household contacts with no symptoms of TB or any other disease at the time of sample collection were subjected to tuberculin skin test. These subjects, with ensured exposure to *M. tb* from A-TB patients (HHC-TB), yet asymptomatic were considered as potential LTB cases. Following WHO's recommendations for high TB-burden low – and middle-income countries, IGRAs was not performed on these individuals as it is not recommended neither for screening nor prediction of LTB to active TB infection. In these countries, it is recommended that IGRAs should not replace TST for the screening of LTB infection [10]. Healthy controls (HC) constituted of clinically healthy volunteers with no reported history of TB or evidence of TB exposure in near past. HC were also subjected to IGRA using QuantiFERON®-TB Gold (QFT®) ELISA kit. For QuantiFERON-TB Gold, results were analysed by QuantiFERON-TB Gold Analysis software (Version 2.62) as per the manufacturer's instructions. Clinical details of the subjects included in the study are summarized in Supplementary Table S1 and S2.

### Sample preparation and ^1^H-NMR acquisition

Serum samples were immediately stored at −80°C after collection. At the time of NMR analysis, samples were thawed on ice. 150 μL of 100% D_2_O buffer (40 mM TSP, 75 mM Na_2_HPO_4_, pH 7.4) were added to 500 μL of serum. Samples were filtered through a centrifugal filter (cut off 10 kDa) to remove macromolecules. After this, 550 μL of the mixture were transferred to a 5-mm NMR tube for analysis. ^1^H-NMR spectra were acquired using a Bruker Avance II 600 MHz spectrometer equipped with a room temperature HCN inverse Z-gradient probe. at 37°C. A standard nuclear overhauser effect spectroscopy (NOESY) experiment [24] was acquired for each sample with a total of 64 accumulations and 72k data points over a spectral width of 20 ppm. A 4-second relaxation delay was included between free induction decays. The water presaturation pulse of 25 Hz was applied throughout the relaxation delays to improve solvent suppression. In addition, for assignment purposes, homonuclear 2D ^1^H-^1^H total correlation spectroscopy and 2D ^1^H, ^13^C heteronuclear single quantum correlation spectra were acquired for selected samples. All spectra were multiplied by a line-broadening factor of 1 Hz and Fourier transformed. Spectra were automatically phased and baseline corrected, chemical shift referenced internally to the methyl group signal of alanine at 1.47 ppm using TOPSPIN 3.0 (Bruker Biospin).

### Multivariate statistical analysis

^1^H-NMR spectra were binned using Amix 3.9.7 (Bruker Biospin) into 0.01-ppm-wide rectangular buckets over the region δ 9.33–0.06 ppm. The residual water signal region (δ 5.20–4.27 ppm) was excluded from the analysis to avoid interference arising from differences in water suppression. All bucket intensities were normalized to the total area of the corresponding spectra. Multivariate statistical analysis was carried out using SIMCA-P v.14.1 software (Umetrics AB, Sweden). Before multivariate analysis, data was Pareto scaled by dividing each variable by the square root of 1/SD, where SD represents the standard deviation value of each variable. Principal Component Analysis (PCA), a non-supervised statistical approach, was performed on normalized and scaled data for identifying potential patterns, intrinsic clusters and outliers. Next, orthogonal partial least square to latent discriminant analysis (OPLS-DA), a supervised statistical approach, was conducted for identifying the variables most relevant for the discrimination between groups compared. The default method of 7-fold internal cross validation was applied, from which R^2^Y (goodness of fit parameter) and Q^2^Y (predictive ability parameter, estimated by cross validation) values were extracted. Those parameters, together with the corresponding permutation tests (*n*=100), were used for the evaluation of the quality of the OPLS-DA models obtained. The variable influence on projection (VIP) list of each OPLS-DA model was inspected and used to identify which NMR signals were important for discriminating between groups. Variables with a VIP value higher than 1 were considered to be relevant for group discrimination. Finally, shared and unique structures plots (SUS-plots) were also obtained to evaluate the shared (metabolites aligned with the diagonals) and unique differences (metabolites aligned with the axes) found when comparing two OPLS-DA statistical models.

### Quantitative analysis of selected metabolites

The variable size bucketing module in Amix 3.9.7 was used to obtain the exact integral value corresponding to the NMR signals identified as relevant for the discrimination. Metabolites of interest were assigned using Bruker NMR Metabolic Profiling Database BBIOREFCODE 2.0.0 database (Bruker Biospin, Rheinstetten, Germany), in combination with other existing public databases [25,26]. The relative change in the levels of metabolites of interest was measured calculating the mean fold change between the groups in the comparison. The statistical significance of the differences between the means of the two groups compared was assessed using the Student *t* test. A *p*-value <0.05 (confidence level 95%) was considered statistically significant.

### Metabolite set enrichment analysis

Metabolite Set Enrichment Analysis (MSEA), based on the analysis of the main metabolites contributing to the discrimination between the groups, was carried out using MetaboAnalyst [27]. Metabolic pathways showing a fold enrichment higher that 1 and a false discovery rate (FDR) < 0.05 were considered significantly altered.

### Logistic regression variable selection

Logistic regression analysis was performed using the forward selection (Likehood Ratio) method in the SPSS version 10.0 software (SPSS, Inc., Chicago, IL, USA). Odds ratio (OR) values were calculated for all the variables included in the equation. A *p*-value < 0.05 (confidence level 95%) was considered statistically significant.

## Results

### Differential metabolic profiles between HC and TB subgroups of patients

**Figure 1.**
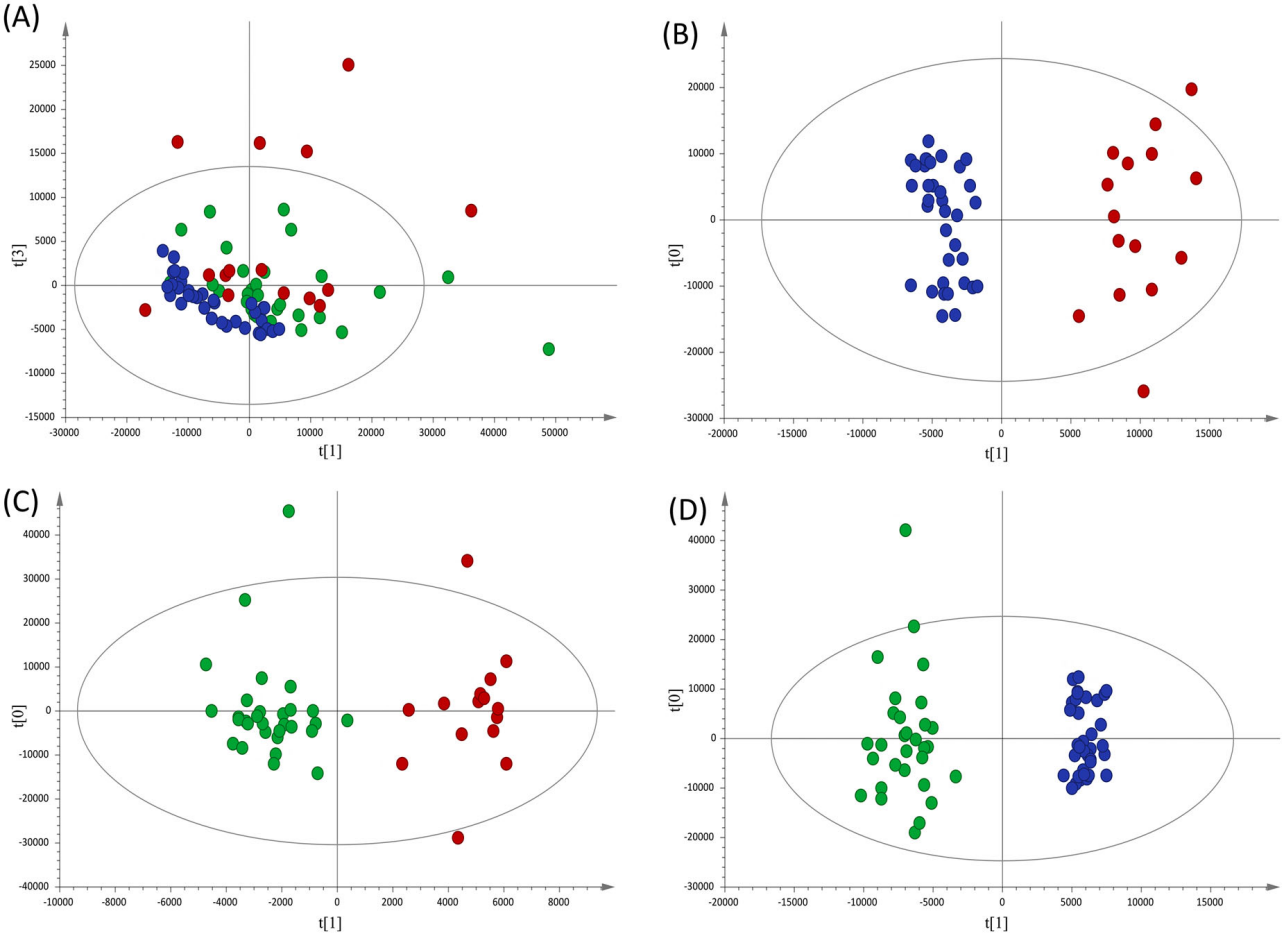
Multivariate statistical analyses of the different clinical groups included in the study. (A) Principal component analysis (PCA) score plots of the healthy controls (HC, blue), house-hold contacts of active TB patients (HHC-TB, green) and active TB patients (A-TB, red) serum samples. Orthogonal partial least squares-discriminate analysis (OPLS-DA) score plots for the comparison between (B) A-TB (red) and HC (blue) (R^2^Y= 0.937, Q^2^Y= 0.893), (C) A-TB (red) and HHC-TB (green) (R^2^Y= 0.900, Q^2^Y= 0.428) and (D) HHC-TB (green) and HC (blue) (R^2^Y= 0.965, Q^2^Y= 0.915).

Non-supervised analysis of the ^1^H-NMR spectra showed a clear distribution of the samples according to disease status (Figure 1A). To further advance in the analysis of the metabolic alterations, discriminant statistical models (OPLS-DA) were built based on the comparisons between the different groups of patients included in the study. These analyses revealed that serum samples from A-TB patients exhibit a specific serum metabolic profile compared with HC (Figure 1B, *R*^2^ = 0.937; *Q*^2^ = 0.893) and with HHC-TB (Figure 1C, *R*^2^ = 0.900; *Q*^2^ = 0.428). A similar analysis performed to compare the serum metabolic profile of HHC-TB and HC subjects (Figure 1D, *R*^2^ = 0.965; *Q*^2^ = 0.915) showed that latent infection has also a reflection in the metabolic profile of patients. Finally, an inspection of the differences in the contribution of each spectral region in the OPLS-DA models for the comparison of HC with A-TB patients and HHC-TB subjects, respectively (Supplementary Figure S1) was conducted. The SUS-plot analysis showed that, although most of the metabolic differences were common when comparing either A-TB patients or HHC-TB subjects with HC, other specific spectral regions were differentially contributing to each model.

### Characterization of the metabolic profile associated with active TB

**Figure 2.**
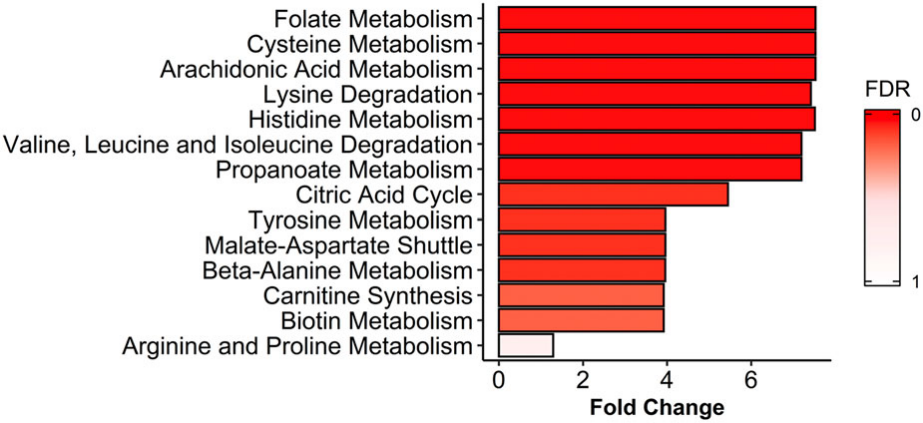
Metabolite set enrichment analysis (MSEA) of differentiating metabolites from A-TB patients and HHC-TB subjects. The horizontal bar graph shows most altered metabolic pathways with fold enrichment higher that 1 (increasing false discovery rate (FDR) values coloured from red to white).

In order to further analyse if the metabolic alterations detected in the serum of A-TB patients were in accordance with what is already known about the biology and the metabolic changes associated with the active TB disease, a MSEA for the identification of the metabolic pathways specifically altered in patients with A-TB, as compared with HHC-TB subjects, was performed (Figure 2). A total of 7, out of the 14 metabolic pathways with a fold enrichment higher than 1, were statistically significant altered (FDR<0.05).

### Differentially altered metabolites in active TB serum samples

**Figure 3.**
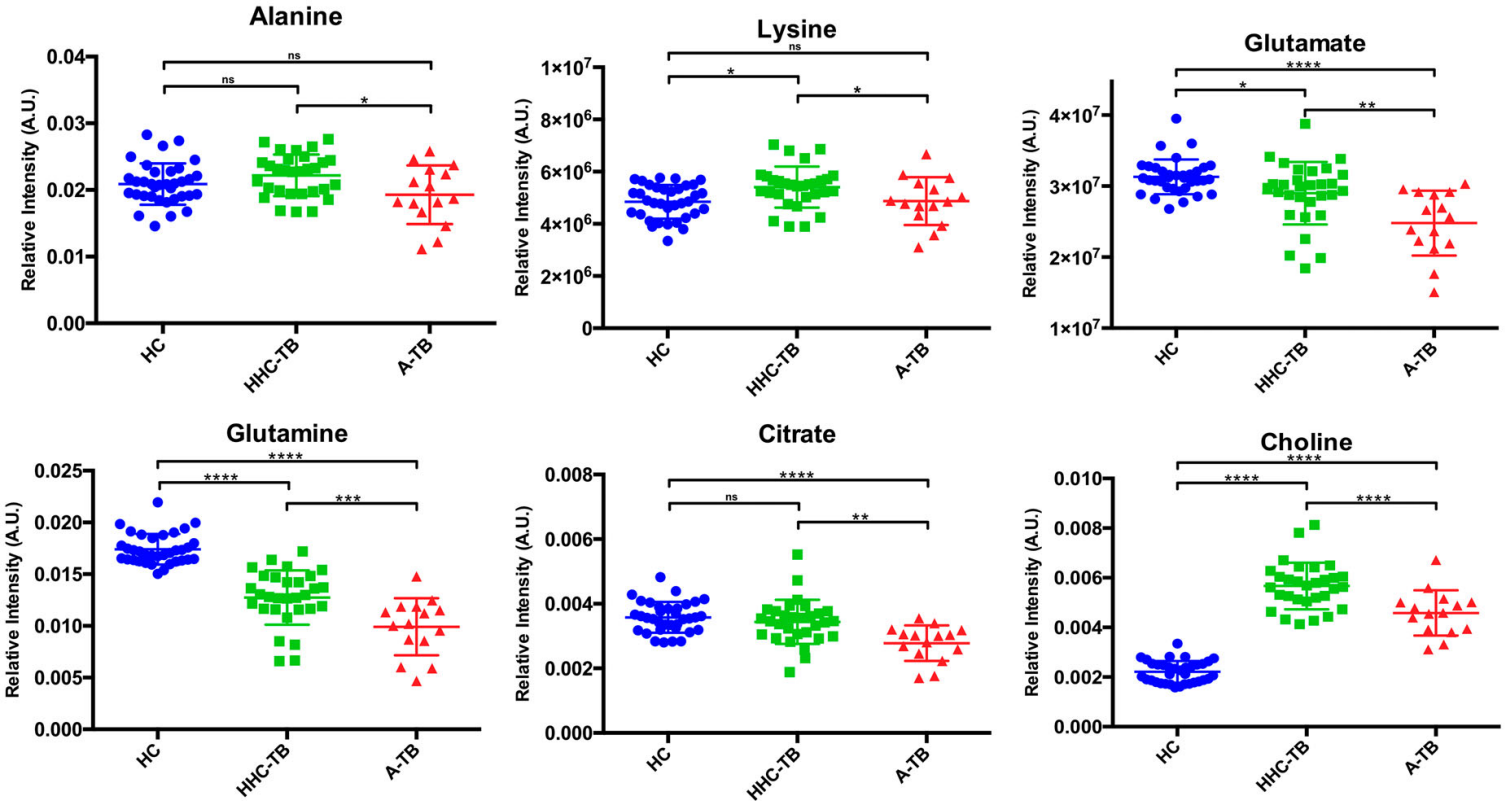
Dot plots showing the relative quantification in the three groups of study (HC, HHC-TB and A-TB) for the metabolites exhibiting statistically significant differences in the comparison between A-TB patients and HHC-TB subjects. NMR signal intensities were normalized to the total area of the spectra. (*p-value < 0.05; ** *p*-value < 0.01; *** *p*-value < 0.001; **** *p*-value < 0.0001; n.s: *p*-value > 0.05).

The analysis of the VIP lists derived from the OPLS-DA models built for the groups of samples included in the study allowed the assignment of the metabolites most relevant for the discrimination between groups. Using this approach, a total of 21 NMR signals were integrated in the NMR spectra and the statistical significance of these changes was evaluated between the different groups of study (Table 1). Compared to HC, the serum metabolic profile of A-TB patients is characterized by statistically significant alterations in the levels of 15 metabolites. Similarly, when comparing HHC-TB with HC subjects, 13 out of these 15 alterations were also statistically significant and presented similar mean fold change values. Interestingly, only differences in the levels of 6 metabolites were detected when comparing the serum metabolic profiles of A-TB patients and HHC-TB subjects. An analysis of the variations in the levels of the 6 metabolities differentiating between A-TB patients and HHC-TB subjects was also carried out in the three groups of the study. Figure 3 shows the relative quantification of the metabolites that showed the most significant variations when comparing A-TB patients and HHC-TB subjects.

**Table 1.** Mean fold changes and *p*-values for relevant metabolites based on the OPLS-DA models for the different comparisons included in the study.

Metabolite	A-TB *vs* HC^a^	*p*-value^c^	HHC-TB *vs* HC^b^	*p*-value^c^	A-TB *vs* HHC-TB^a^	*p*-value^c^
Valine	0.960	0.390	1.039	0.275	0.924	0.120
Propylene glycol	**1.811**	**0.001**	1.692	0.097	1.071	0.860
Lactate	**1.340**	**0.023**	**1.477**	**0.005**	0.907	0.629
Alanine	0.999	0.989	**1.212**	**0.001**	**0.825**	**0.028**
Lysine	1.005	0.911	**1.117**	**0.002**	**0.900**	**0.046**
2-Aminobutyrate	**1.110**	**0.026**	**1.154**	**1.87E-04**	0.962	0.469
Acetate	**1.358**	**0.017**	**1.496**	**4.47E-04**	0.908	0.559
Glutamate	**0.792**	**3.47E-08**	**0.926**	**0.010**	**0.855**	**0.005**
Glutamine	**0.610**	**2.39E-13**	**0.810**	**5.45E-07**	**0.753**	**2.03E-04**
Citrate	0.932	0.203	**1.129**	**0.023**	**0.825**	**0.018**
Methionine	**0.872**	**0.001**	0.926	0.070	0.943	0.411
Choline	**2.263**	**1.91E-14**	**2.928**	**9.41E-19**	**0.773**	**0.012**
Myo-inositol	**1.128**	**0.034**	**1.093**	**0.024**	1.032	0.639
Proline	**0.899**	**0.025**	**0.852**	**2.16E-05**	1.056	0.479
Aspartate	**0.903**	**0.0114**	**0.853**	**9.26E-06**	1.059	0.396
Creatine	**0.859**	**0.005**	**0.909**	**0.033**	0.945	0.433
Asparagine	**0.779**	**0.004**	**0.771**	**6.60E-05**	1.010	0.931
Glucose	1.017	0.834	0.936	0.315	1.086	0.408
Histidine	**0.715**	**0.024**	**0.770**	**0.022**	0.929	0.653
Phenylalanine	**1.661**	**3.89E-13**	**1.563**	**2.55E-11**	1.063	0.426
Tryptophan	0.932	0.134	1.034	0.480	0.901	0.217

Bold values indicate significance (*p*-value < 0.05).

^a^Underlined values indicate metabolites with higher levels in A-TB patients' serum.

^b^Underlined values indicate metabolites with higher levels in HHC-TB subjects' serum.

^c^Student *t* test.

### Regression model describing TB stage

**Figure 4.**
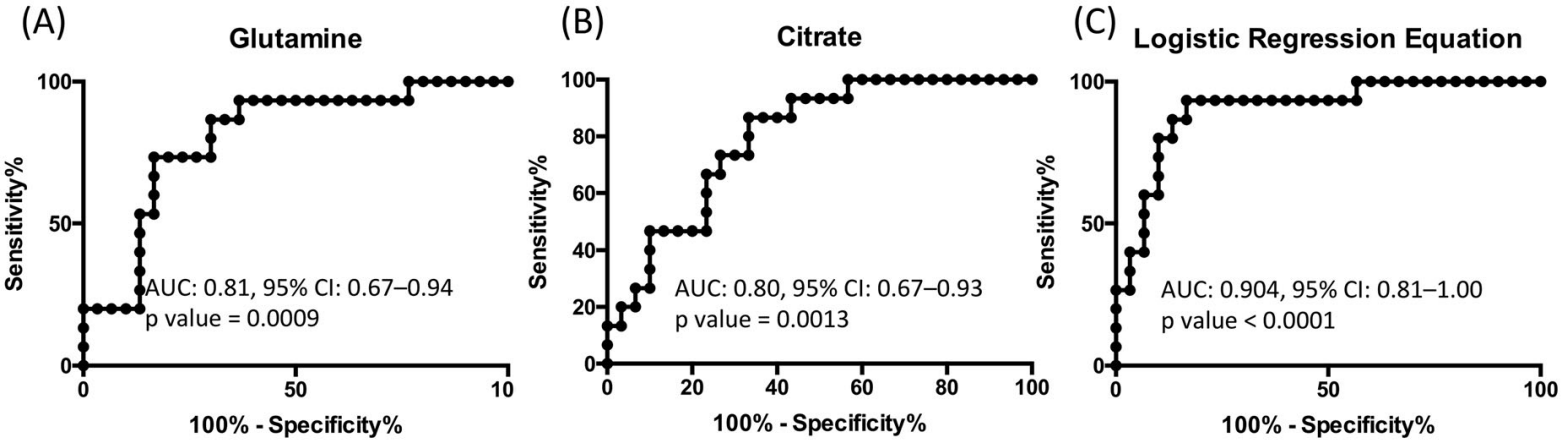
Receiver operating characteristic model of the metabolites included in the logistic regression equation: (A) Glutamine and (B) Citrate, and (C) the logistic regression equation obtained for the discrimination between HHC-TB and A-TB patients.

In order to further advance in the clinical potential of the metabolite alterations identified in this study, a logistic regression analysis of the data was performed. To this end, metabolites whose levels experienced statistically significant changes when comparing HHC-TB and A-TB patients were evaluated to generate a logistic regression equation. Using this approach, characteristic lower levels of glutamine and citrate were found to be predictive of active TB in serum samples from A-TB and HHC-TB patients (Table 2). Internal validation of the logistic regression equation was performed by evaluating the area under the curve (AUC) values of the receiver operating characteristic (ROC) curves for each individual metabolite included in the equation and for the logistic regression equation (Figure 4).

**Table 2.** Characteristics of the logistic regression equation obtained for the discrimination between HHC-TB subjects and A-TB patients.

Metabolite	β^a^	OR^b^	1/OR	*p*-value
Glutamine	−0.072	0.931	1.074	0.004*
Citrate	−0.0232	0.793	1.261	0.016*
Constant	15.695	6.55E+06	1.53E-07	0.003*

^a^β: Coefficient of logistic regression.

^b^OR: odds ratio.

*Statistically significant (*p*-value < 0.05).

## Discussion

Effective TB disease management would require not only identifying active TB cases at an early stage, but also TB exposed asymptomatic individuals who are potential cases of latent TB. Efforts to identify TB specific biomarkers that could help to better understand disease pathogenesis and to effectively distinguish patients from healthy and latent asymptomatic cases remain a fundamental goal in this area [28]. In this context, this study represents the first application of high field ^1^H-NMR metabolomics, based on the analysis of a significant number of samples, to characterize and compare the specific serum metabolic profiles of active TB patients and asymptomatic subjects with definite infection by *M. tb* due to close proximity to active TB patients. This metabolomics study reveals that both A-TB patients and HHC-TB subjects show a specific serum metabolic profile when compared to HC. Thus, statistically significant changes in a specific set of metabolites were shared when comparing either HHC-TB subjects or A-TB patients to clinically HC, which could point to potential asymptomatic latent cases, based on their confirmed recent TB exposure. Even though there were a number of common metabolic changes between these two comparisons, the results showed that there also exist metabolic alterations specifically associated with each stage of the disease.

The analysis of the variations in the intensities of all the metabolites playing an important role in the discrimination models revealed 14 pathways being significantly altered between A-TB patients and HHC-TB subjects. Interestingly, the three top ranked pathways in this analysis are known TB metabolic-related pathways (folate, cysteine and arachidonic acid metabolism), thus confirming the validity of our findings [29–31].

The potential of the folic acid biosynthesis pathway as a target for the development of antimicrobial agents has been acknowledged for many years [29]. In particular, the second line anti-TB drug, para-aminosalicylic acid (PAS), specifically blocks growth of *M. tb* when its active forms inhibit dihydrofolate reductase activity, an essential enzyme in folate metabolism [32,33]. Cysteine biosynthesis pathway has also an important role in defense against oxidative stress in *M. tb*. The redox active sulfhydryl group of mycothiol, the functional analog to glutathione in mycobacteria, is directly derived from L-cysteine. Mycothiol is used by mycobacteria to maintain an intracellular reducing environment and to protect against cellular damage from oxidative species and xenobiotics [34–36]. In this context, results from a recent metabolomics study in *M. tb* suggested perturbations in cysteine metabolism under microbicidal stresses [37]. Finally, it has been reported that free unsaturated fatty acids, including arachidonic acid, can stimulate the activation of dormant *M. tb* cells in liquid medium [30]. Moreover, eicosanoids, lipid mediators derived from arachidonic acid, have been associated with the modulation of the host response to *M. tb* infection [38,39]. In fact, published studies have also reported increased eicosanoid ratios in plasma in A-TB patients compared to HHC-TB or HC subjects [40].

After having determined that the differences observed in our study in the serum metabolic profile of different stages TB patients were in accordance with what is already known about metabolic changes involved in the progression of the disease, the specific metabolic changes between HHC-TB and A-TB patients were further analysed. The major differences in serum metabolite levels detected between HHC-TB and A-TB patients included decreased levels of aminoacids such as alanine, lysine, glutamate and glutamine, and citrate and choline in patients with active infection. In a recently published study [41], amino acid uptake analyses indicated that glutamine, glutamate and alanine are taken up and rapidly metabolized by *M. tb* as nitrogen sources. Moreover, alanine dehydrogenase, an enzyme playing an essential role in alanine utilization as a nitrogen source, has been implicated in adaptation of *M. tb* to anaerobic dormant stage in LTB and has been reported to be a useful tool to discriminate between active TB and LTB [42,43]. Similarly, glutamine synthetase, an enzyme that plays a key role in both nitrogen metabolism and cell wall biosynthesis, has been investigated as a novel antibiotic strategy [44] Additionally, citrate lyase (CitE) activity has also been shown to be indispensable for *M. tb* pathogenesis *in vivo* [45]. In a ^13^C-based metabolomic profiling study, it was demonstrated that *M. tb* slows and remodels its tricarboxylic acid cycle to increase production of succinate from isocitrate [46]. This remodelling is mediated by the bifunctional enzyme isocitrate lyase. Thus, isocitrate lyase-dependent production of succinate affords *M. tb* with a unique and bioenergetically efficient metabolic means of entry into and exit from hypoxia-induced quiescence [46]. Interestingly, decreased citrate levels were found in our study in the serum of TB patients, perhaps reflecting the increase in citrate uptake by *M. tb* or *M. tb* infected cells. Deviation of plasma citrate levels from homeostatic concentrations (normal plasma levels) has been linked to various physiological changes and clinical consequences that may directly or indirectly impair immunity [47].

In a previous ^1^H-NMR metabolomics study, Zhou *et al*. [48] observed higher levels of lysine in serum samples of A-TB patients when compared to HC. No statistically significant differences in the levels of lysine were found when comparing A-TB patients and HC in our study. However, it was found that this metabolite exhibits statistically significant decreased levels when comparing HHC-TB and A-TB patients. These results are in agreement with previous results obtained from the analysis of TB-infected lung tissue samples [23]. Somashekar *et al*. [49] applied a ^1^H-NMR metabolomics approach using lung and serum samples collected from guinea pigs infected with *M. tb*. This study, accordingly with our results, showed that choline-containing compounds were detected in reduced amounts in the serum of the infected animals when compared with naïve controls.

Finally, in an effort to evaluate the clinical potential of the metabolite alterations identified in this study for the discrimination between HHC-TB and A-TB patients, a logistic regression analysis was performed. This equation, describing a specific combination of two metabolites, enabled the discrimination between HHC-TB and A-TB patients with AUC value of 0.904 (confidence interval 0.81-1.00, *p*-value < 0.0001). These results suggest glutamine and citrate as potential metabolic biomarkers indicating *M.tb* active infection. In a recent study, involving a cohort of TB-exposed individuals across Subsaharan Africa [50], Weiner *et al*. described a specific metabolic biosignature for TB enabling the identification of future progressors. Similar to our observations, they observed a decrease in serum levels of glutamine in TB-exposed individuals several months prior to clinical TB. Together, these two studies strengthen the potential of metabolite profiling as a sensitive tool for biomarker search in TB control. In this context, our work represents the first example, in an Indian cohort of TB patients, on how non-invasive serum biomarkers able to distinguish active TB or potential LTB cases can be identified based on the metabolic differences between A-TB patients and HHC-TB individuals. In the last few years, enrolment of HHC-TB individuals in TB studies, specifically focused on the identification of potential LTB cases is gaining importance [3,4]. Thus, with no recommended diagnostics that can ensure *M.tb*.-exposure, HHC-TB included in this study were surely *M.tb*.-exposed, irrespective of their TST responses. Furthermore, as HHC-TB individuals were close relatives of A-TB patients and resided with them, variations in metabolic profile due to habitat divergence were also minimized.

Although further validation of the results, in independent cohorts and comparison with other pulmonary infectious diseases, will be necessary to increase the robustness of this analysis, our data support the idea that the characterization of a specific metabolic profile associated with active TB infection holds great promise in the identification and development of new serum biomarkers for the diagnosis of this disease.

Overall, this study aimed at a better understanding, both in terms of magnitude and change direction, of the metabolic alterations responsible for the progression of TB infection. This information could be specifically useful for immune-compromised population, where prophylactic treatment can be initiated if TB exposure could be diagnosed.

Finally, the strategy described in this work provides a sensitive, specific, and minimally invasive method for the identification of potential metabolic biomarkers that may aid in the early diagnosis and staging of TB and in the optimization of current risk stratification models.

## Supplementary Material

Supplemental Material
