# Supplementary figures and images for: Active and prospective latent tuberculosis are associated with different metabolomic profiles: clinical potential for the identification of rapid and non-invasive biomarkers
Source: Emerg Microbes Infect. 2020 Jun 2;9(1):1131–9. doi: 10.1080/22221751.2020.1760734 (PMC7448900; doi:10.1080/22221751.2020.1760734)

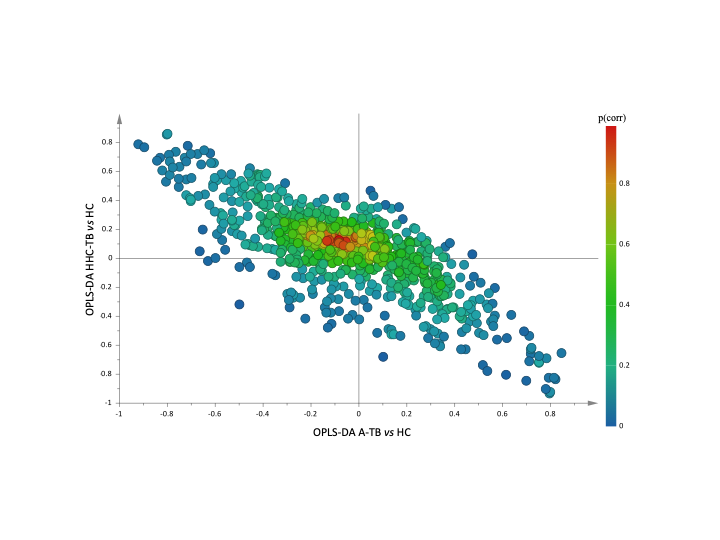

Supplement: Supplemental Material [file TEMI_A_1760734_SM4890.tiff]
